# Efficacy of VG26 and K1 acupuncture points in cardiopulmonary resuscitation of neonatal puppies born by cesarean section

**DOI:** 10.3389/fvets.2025.1579758

**Published:** 2025-04-09

**Authors:** Maria Lucia G. Lourenço, Fabíola C. Knupp, Keylla Helena N. P. Pereira, Kárita M. Fuchs, Gleice M. Xavier, Júlia C. Mendonça, Miriam Harumi Tsunemi, Fabiana F. Souza, Jean G. F. Joaquim

**Affiliations:** ^1^Veterinary Neonatology Research Group, Department of Veterinary Clinics, School of Veterinary Medicine and Animal Science, São Paulo State University (UNESP), Botucatu, Brazil; ^2^Department of Veterinary Medicine, Federal University of Alagoas (UFAL), Viçosa, Brazil; ^3^Institute of Biosciences, São Paulo State University (UNESP), Botucatu, Brazil; ^4^Department of Veterinary Surgery and Animal Reproduction, School of Veterinary Medicine and Animal Science, São Paulo State University (UNESP), Botucatu, Brazil

**Keywords:** neonatology, score Apgar, acupuncture, cesarean section, cardiopulmonary resuscitation

## Abstract

The aim of this study was to evaluate the effects of acupuncture points VG26 and K1 on the reversal of apnea and the elevation of heart and respiratory rates in neonatal dogs after birth. Sixty neonates who were apneic, bradycardic, bradypneic or dyspneic were divided into three groups: VG26 acupuncture point (GVG26), K1 acupuncture point (GK1), and control (CG). Data on heart rate (HR), respiratory rate (RR) and Apgar score were collected. The moments of analysis were after 1 min of tactile respiratory stimulus (M1); after stimulation with the acupuncture points (M2); and 10 min after the last assessment (M3). After the VG26 and K1 were used, a significant increase in HR was observed between M1 and M2, medians (25% quartile–75% quartile), respectively: GVG26 136 and 202 bpm; GK1 147 and 195 bpm; GC 168 and 187 bpm. Significant clinical improvement was also observed in RR, with a median at M1 and M2, respectively: GVG26 16 and 28 mpm; GK1 18 and 32 mpm; GC 32 and 40 mpm. For the Apgar score, significant differences were observed in the mean scores between M1 and M3 in the GVG26, GK1 and CG groups. The VG26 and K1 acupuncture points are effective in neonatal puppies, leading to improvements in the respiratory pattern and increases in the HR, RR and Apgar score, can be used in neonatal resuscitation at birth.

## 1 Introduction

The canine neonatal mortality rate in veterinary medicine varies from 17 to 30%, being highest during birth and in the first hours of life. Birth is a critical period of adaptation to extrauterine life and a great challenge for neonatal survival. Among the various physiological adaptations, newborns must maintain adequate pulmonary breathing and heart rate, ensuring the efficiency of gas exchange, cardiac output and tissue perfusion (1–3).

Although most neonates transition to extrauterine life without the need for intervention, many need help to begin breathing, and a small number are referred for advanced resuscitation maneuvers (4, 5). These interventions are often necessary in cases of dystocia, prolonged labor and cesarean sections, as these situations lead to varying degrees of neurological clinical depression, apnea or respiratory distress and bradycardia (3, 4, 6, 7). Therefore, it is important to have a team trained in neonatal care procedures during cesarean section, ensuring immediate intervention and survival of the litter.

Acupuncture is a technique derived from traditional Chinese medicine that consists of inserting a filiform needle into specific skin points, called acupoints, with the purpose of triggering physiological stimuli capable of controlling pain and/or maintaining the functionality of the autonomic nervous system (8, 9).

What defines an acupoint histologically is the large concentration of nerve endings (afferent fibers) and other nerve receptors found in the skin, low electrical resistance that generates greater electrical conductance and the presence of a large amount of ions. Stimulation of acupoints leads to the release of mast cells, histamine and calcium, thus stimulating nerve receptors and free nerve endings, as well as capillaries in the region. Thus, there are viscera-somatic and somato-visceral reflexes depending on the region and location of the stimulated acupoint (9–11).

The vasogoverning acupoint VG26 (Renghong) can be used in cardiopulmonary resuscitation, shock, coma, syncope, epilepsy, collapse, anesthetic apnea, and circulatory and respiratory stimulation of newborns and, when stimulated, results in increased CNS activity, increasing respiratory and cardiac frequencies, in addition to being a point of significant release of endorphins (12–14). The K1 acupoint (Yongquan) also known in acupuncture as the “kidney 1 point” is indicated for use in coma, status epilepticus, anesthetic recovery, increased heart rate and other vital signs, pharyngitis, constipation, dysphonia, dysuria and urinary incontinence (15, 16).

Despite the existence of studies on the use of the VG26 acupuncture point in cases of respiratory depression or apnea in dogs and cats during the induction or maintenance of general anesthesia (12, 13, 17, 18), and reviews on neonatal resuscitation in dogs at birth that mention the use of the point (4, 5, 19–21), there are no clinical studies that demonstrate the effectiveness of VG26 in neonatal resuscitation of dogs at birth. Furthermore, few studies have investigated the use of the K1 point in dogs. Therefore, the aim of this study was to evaluate the effects of acupuncture points VG26 and K1 on the reversal of apnea and the elevation of heart and respiratory rates in neonatal dogs after birth. We hypothesize that acupuncture points VG26 and K1 provide clinical improvement in apneic, bradypneic or bradycardic patients.

## 2 Materials and methods

This study was carried out by the Veterinary Neonatology Research Group of the Faculty of Veterinary Medicine and Zootechnics of the Universidade Estadual Paulista (Unesp), Botucatu, Brazil. The study included 60 neonatal puppies of diverse breeds [English Mastiff (5), French Bulldog (21), Miniature Pischer (6), American bully (7), Pitbull (4), German Spitz (4) and mixed breed (13)] from cesarean sections due to maternal or fetal dystocia treated at the Small Animal Clinical and Reproduction Service of the Veterinary Hospital of FMVZ Unesp. The animals were included in the study only after approval by the Ethics Committee on the Use of Animals, protocol number 0518/2023, and authorization of the owners by signing the free and informed consent form.

The inclusion criteria for the study were full-term neonates born by emergency cesarean section (maternal or fetal dystocia) from clinically healthy bitches. The exclusion criteria for this study were newborns who presented congenital malformations (hydrocephalus, cleft palate, pectus excavatum), infections or other changes and diseases (sepsis, parasitic disease, prematurity, low weight).

### 2.1 Procedures and assessments

The study included bitches aged between 1 and 7 years, with adequate body score, dewormed and vaccinated, who were sent to a cesarean section showing signs of dystocia, such as uterine atony, incarceration of the puppy in the birth canal due to size or fetal statics, narrowing of the birth canal assessed by pelvimetry, or fetal distress assessed by ultrasound.

The anesthetic protocol for cesarean sections was performed with induction with intravenous propofol at a dose sufficient to cause loss of the laryngotracheal reflex. Then, epidural anesthesia was performed with 2% lidocaine, 4 mg/kg, single dose. Anesthetic maintenance was performed with 1% of isoflurane diluted in 100% oxygen, which was administered through a circular valve anesthetic circuit. The expired tidal volume was 10–15 mL/kg, minute ventilation was 1.5 L/min/10 kg, and oxygen flow was 0.5–1 L/min/10 kg. After removing the puppies from the uterus, fentanyl (5 μg/kg, single dose) was administered intravenously to the bitches.

**Figure 1:**
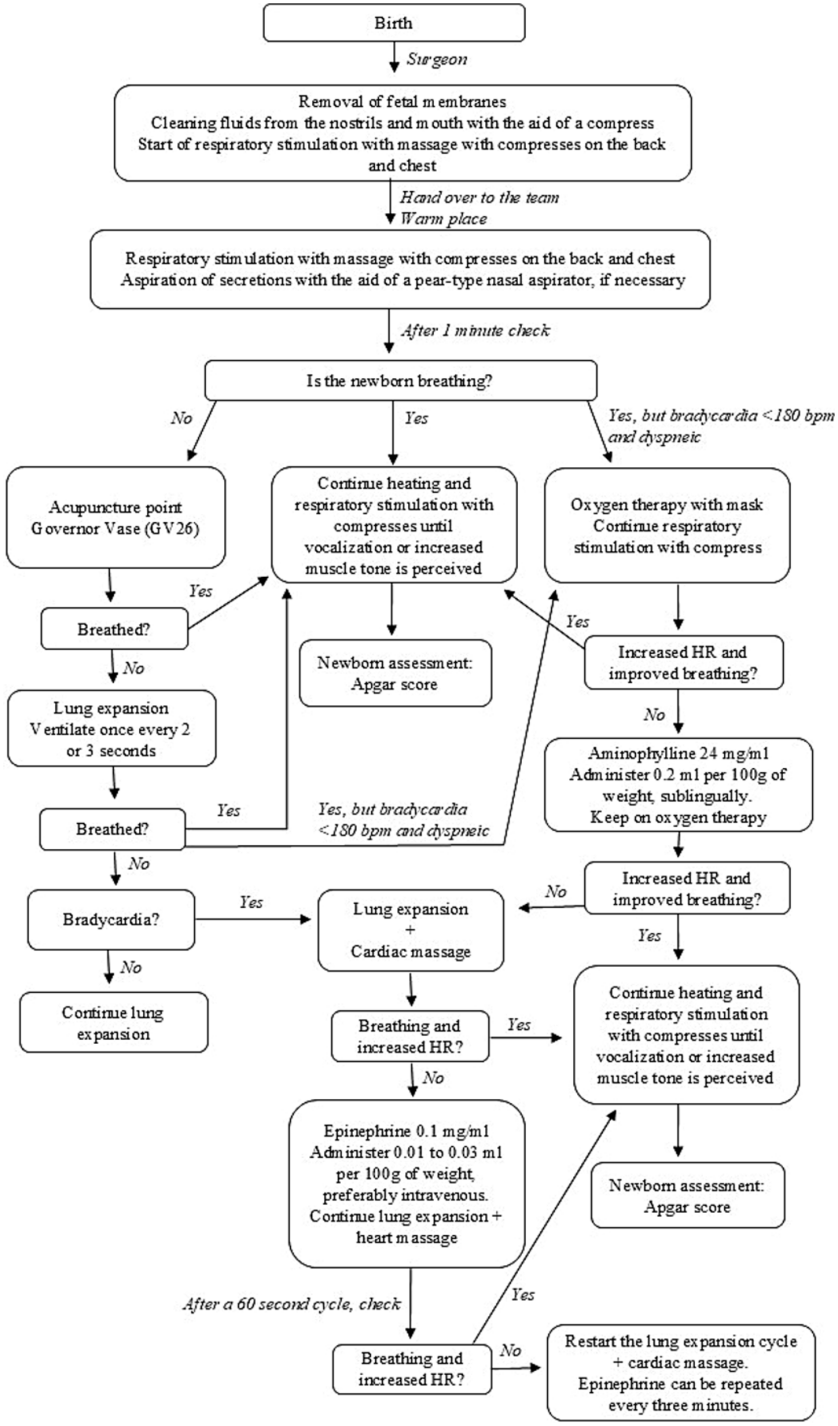
Flowchart describing step-by-step maneuvers to stimulate breathing and cardiopulmonary resuscitation of neonatal dogs at birth. Adapted from Faiz et al. (16).

The respiratory stimulation and resuscitation protocol at birth was used according to the flowchart described in Figure 1. After the puppies were removed from the uterus and removing the fetal coverings, tactile stimuli were performed, such as massage in the thoracic region with the aid of compresses, as stimulus initial respiratory, with puppies expected to breathe spontaneously within 1 min. After this stimulus, to determine the clinical condition and neonatal vitality at birth, the puppies were evaluated for heart rate (HR), respiratory rate (RR), respiratory pattern, and Apgar score.

Sixty apneic, bradypneic (<15 bpm), dyspneic or bradycardic (<180 bpm) newborns were randomly assigned to the following groups: stimulation of acupuncture point VG26 (GVG26) *n* = 20, stimulation of acupuncture point K1 (GK1) *n* = 20, and carrying out conventional resuscitation procedures (positive pressure ventilation, mask oxygen therapy, cardiac massage and/or administration of epinephrine) – control group (CG) *n* = 20.

The moments for evaluating the HR, RR and respiratory pattern were as follows: M1 – after 1 min of tactile respiratory stimuli with the aid of a compress and drying of the puppies; M2 – after stimulation with the acupuncture points (rapid pistoning movements of the acupoints for 30 s) or conventional maneuvers in the CG (for 30 s); and M3–10 min after the last assessment, to compare the evolution of neonatal parameters between the groups. The Apgar score was assessed at M1 and M3.

The Apgar score was used to assess mucous membrane color, HR, RR, muscle tone and reflex irritability. The heart rate was assessed via a stethoscope on the left side of the chest, and the respiratory rate was measured by observing chest expansion movements. Muscle tone was determined with the neonate in the supine position, observing active movements of the limbs. The irritability reflex was assessed by painful stimulus pressing on the interdigital space. Mucosal color was assessed by visualizing the oral mucosa. Each parameter received a score of 0, 1 or 2, according to what was presented by the neonate (Table 1), and the sum identified neonatal vitality. Score interpretation was assessed by scoring 0–3 for weak vitality, 4–6 for moderate vitality and 7–10 for normal vitality (2, 7).

**Table 1:** Modified Apgar score for neonatal dogs.

		Score	
Parameters evaluated	0	1	2
Mucous membrane staining	Cyanotic	Pale	Rosea
Heart rate	<100 bpm	<200 bpm	200–260 bpm
Respiratory rate	Absent <6 mpm	Weak and irregular <15 mpm (6–15)	Regular and rhythmic >15 mpm
Muscle tone	Flabby	Some flexion member	Flexion
Reflex irritability	Absent	Some movement	Crying

Adapted from Veronesi et al. and Vassalo et al. (2, 7). Bpm, beats per minute; mpm, movements per minute.

**Figure 2:**
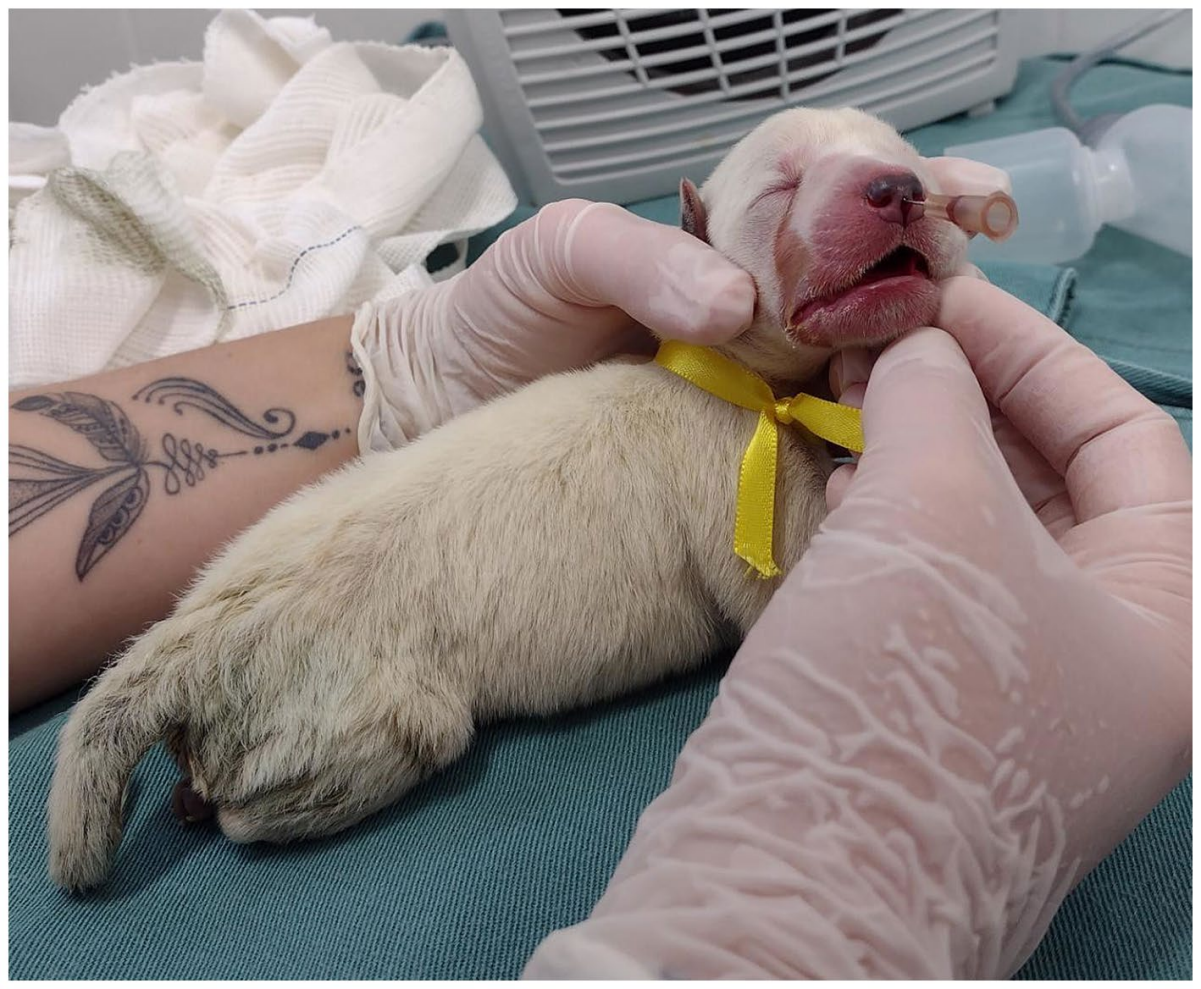
Demonstration of needling and the anatomical location of the VG26 acupuncture point.

**Figure 3:**
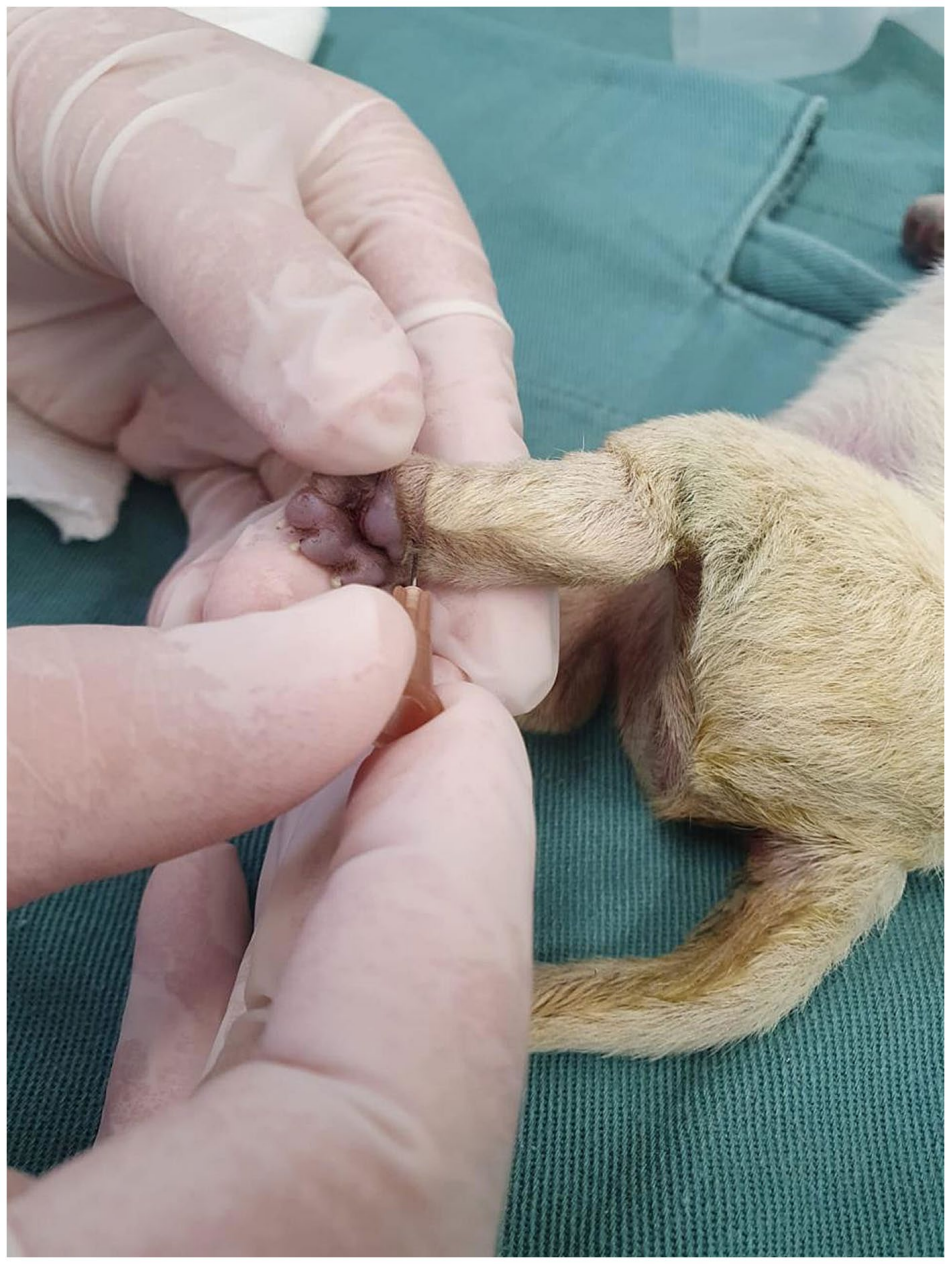
Demonstration of needling at the K1 acupuncture point on the plantar surface.

The acupuncture points were performed using a 0.45 × 13 mm hypodermic needle, with the insertion of 1/3 of the needle length and rapid pistoning movements. The VG26 point was made in the depression of the nasal philtrum at the level of the ventral edge of the nostril (Figure 2; Video 1). The K1 point was made on the plantar surface of the pelvic limb, between the third and fourth metatarsals, below the central cushion (Figure 3; Video 2). In the CG, conventional resuscitation maneuvers were performed, such as positive pressure ventilation using the One Puff Puppy^®^ lung expander, mask oxygen therapy, cardiac massage and/or intravenous administration of epinephrine (0.1 mg/mL) through the jugular vein, were performed, following the resuscitation flowchart (Figure 1).

### 2.2 Statistical analysis

The sample size (*n* = 60) was determined by the ANOVA test, assuming a statistical power of 80%. The normality of the distribution of variables was assessed using the Shapiro–Wilk test. Comparisons of the medians of clinical parameters (HR and RR) between time points within each group were performed using the Friedmann nonparametric test. Comparisons of medians of clinical parameters (HR and RR) between groups were performed using the nonparametric Kruskal–Wallis test. To evaluate and compare the Apgar scores between the time points in each group, the nonparametric Wilcoxon test was used. Spearman’s correlation was performed between the variables (HR, RR and Apgar score). All tests were performed at the 5% significance level (*p* < 0.05).

## 3 Results

The parameters HR and RR differed significantly (*p* < 0.001) between M1 and M2, observing neonatal clinical improvement in M2 after the use of the acupuncture points (Table 2). The median (25% quartile – 75% quartile) HRs in M1 and M2 were, respectively: GVG26 136 and 202 bpm; and GK1 147 and 195 bpm. The median RRs at moments M1 and M2 were, respectively: GVG26 16 and 28 mpm; GK1 18 and 32 mpm. There were no significant differences in the HR and RR between M2 and M3 in the two groups. In GVG26 and GK1, four puppies were in apnea in M1 (two in each group), started breathing between 10 and 30 s after stimulation of the acupuncture points. In the CG, three puppies were apneic in M1 and started breathing between 10 and 30 s after using positive pressure ventilation.

**Table 2:** Median (25% quartile – 75% quartile) heart rate (HR) and respiratory rate (RR) of newborns at the different time points evaluated in each group and between groups.

Parameters	Groups	Moments			
		M1	M2	M3	Value p*
HR (bpm)	GVG26	136 (123–164)^aB^	202 (187-236)bB	210 (192-240)bB	<0.001
	GK1	147 (124-162)aB	195 (187-201)bB	198 (190-216)bB	<0.001
	GC	168 (160-173)aA	187 (179–200) bB	196 (188-203)bB	<0.001
	Value p*	0.0091	0.1307	0.3622	
RR (mpm)	GVG26	16 (11–20) aB	28 (25-31)bB	30 (28-34)bB	<0.001
	GK1	18 (11–28) aA	32 (30–35) bA	36 (30–38) bA	<0.001
	GC	32 (23–40) aA	40 (35–42) bA	40 (36–46) bA	0.02
	Value p*	<0.0001	0.0000	0.0004	

^a,b^ Different lowercase letters indicate significant differences between assessment moments within the same group (*p* < 0.05).

^A,B^ Different capital letters indicate significant differences between groups at the same assessment moment (*p* < 0.05).

The CG, which received conventional resuscitation maneuvers, also demonstrated significant clinical improvement in HR (*p* < 0.001) and RR (*p* < 0.05) between M1 and M2, respectively: CG 168 and 187 bpm; 32 and 40 mpm. There were no significant differences in HR and RR between M2 and M3 (Table 2).

No significant differences were observed between the GVG26, GK1 and CG groups in M2 and M3 (after the use of acupuncture points or conventional resuscitation maneuvers), indicating that the neonatal responses to the use of the VG26 and K1 acupuncture points did not differ from each other, and both lead to clinical improvement and stabilization of HR and RR, as well as these acupoints are effective in the same way as other resuscitation procedures are and can be used in assistance immediate neonatal.

However, in the clinical observation of the GC, which received conventional resuscitation maneuvers (positive pressure ventilation and mask oxygen therapy), in seven of the 20 animals, it was necessary to use advanced resuscitation maneuvers (cardiac massage and the use of epinephrine), but there was no need for other resuscitation procedures in groups VG26 and K1. Demonstrating that starting neonatal resuscitation with acupuncture points can be more effective, avoiding the need to use other resuscitation maneuvers.

In the Apgar score, significant differences (*p* < 0.05) were observed in the mean scores between M1 and M3 in the GVG26, GK1 and CG groups, respectively: 3.3 ± 1.45 and 6.5 ± 1.02; 3.3 ± 1.13 and 7.5 ± 1.04; and 2.8 ± 1.01 and 7.8 ± 0.83. Demonstrating evolution of neonatal vitality after the procedures performed (Table 3).

**Table 3:** Means and standard deviations of the Apgar scores at M1 and M3 (*p* < 0.05).

Groups	Apgar M1	Apgar M3
VG26	3.3 ± 1.45	6.75 ± 1.02
GK1	3.3 ± 1.13	7.85 ± 1.04
GC	2.8 ± 1.01	7.8 ± 0.83

The average weights of the newborns evaluated in each group were 222.85 ± 41 (GV26), 188.00 ± 20 (GK1), and 255.25 ± 106 (CG). There was no mortality in the animals included in the study.

At M1 of VG26, there was a positive correlation (*p* < 0.05) between HR and Apgar score, with correlation coefficients of 0.4944, respectively. At M3 of the VG26, R1 and GC groups, there was a positive correlation (*p* < 0.05) between HR and Apgar score, with correlation coefficients of 0.5532, 0.5508, and 0.5831, respectively.

## 4 Discussion

Despite the lack of clinical studies on the use of acupuncture points in neonatal dogs, review articles on resuscitation maneuvers in puppies at birth recommend the use of the governor vaso acupuncture point (VG26) in cases of apnea, dyspnea, bradypnea or neonatal bradycardia, and if there is no clinical improvement, conventional resuscitation procedures are performed immediately, such as positive pressure ventilation, mask oxygen therapy, cardiac massage or the use of epinephrine (4, 5, 19–21). Acupuncture in conjunction with conventional resuscitation is generally recommended, but acupuncture alone can be used when there is no equipment or drugs available for assistance (13). This study demonstrated that the VG26 and K1 acupoints are effective in neonatal resuscitation, increasing HR and RR, improving the respiratory pattern, and reversing apnea, reducing the need for other resuscitation maneuvers.

In studies with adult dogs in cardiorespiratory arrest, vital parameters returned in approximately 67% of cases in which acupuncture was applied at the VG26 point (13, 17). In newborns, there is a case report in kittens that demonstrated the effectiveness of VG26 in resuscitation after birth (18). There are no studies on the use of the K1 point in the resuscitation of dogs. In humans, clinical trials that performed K1 stimulation after basic and advanced cardiopulmonary resuscitation failed resulted in an approximately 85% survival rate, indicating that the K1 resuscitation maneuver should be formally included in the cardiopulmonary resuscitation sequence protocol (14, 15). In the present study with neonatal dogs, the use of VG26 and K1 led to increases in the HR and RR in 100% of the cases. Despite the neonatal clinical improvement observed after the use of acupuncture points, our study has limitations, since no assessments were performed that could provide important information about perinatal hypoxia and the improvement in oxygenation and tissue perfusion, such as venous blood gas analysis, lactate, glucose, peripheral oxygen saturation and presence of meconium.

These acupoints have sympathomimetic effects on cardiac, circulatory and respiratory system functions (13, 14, 22). The stimulus time described in the literature varies depending on the severity of the cases. When VG26 was needed in 69 cases of respiratory depression or apnea in dogs and cats during the induction or maintenance of general anesthesia, breathing was restored to normal or near-normal rates in 100% of the cases, within 10–30 s of needle insertion. In seven cases of anesthetic apnea with concomitant cardiac arrest and the absence of vital signs, the recovery rate was 43%, and those who recovered required four to 10 min of acupuncture stimulation (12). In a report of 30 cases of human patients hospitalized for different conditions, classified as scoring 3 on the Glasgow Coma Scale, with the absence of vital signs and no pulse, the K1 point was pressed for an average of 3 to 8 min, bilaterally, with an increase in HR and other vital signs observed, as well as a return to or improvement in consciousness (15). In this study, in cases of apnea, bradypnea and bradycardia resulting from anesthetic depression or perinatal asphyxia and consequent hypoxia, it was observed that breathing began within 10–30 s, and the HR and RR increased within 30 s of needling stimulation.

The Apgar score revealed the evolution of neonatal vitality and viability in all the groups studied, with normalization of heart and respiratory rates, improvement in the color of cyanotic to pink mucous membranes, a better response to the stimulus of reflex irritability, and evolution from absent/weak to strong muscle tone, demonstrating the effectiveness of using points in resuscitation, reducing neurological depression and increasing neonatal vitality. The Apgar score is an assessment index routinely used in medicine and veterinary medicine to facilitate clinical assessment at the time of birth, to immediately identify the clinical condition of the neonate at birth, to direct interventions or to monitor the effectiveness of resuscitation maneuvers (2, 3, 7, 23).

Cardiopulmonary resuscitation is considered an emergency procedure that helps reverse cardiorespiratory changes and the state of hypoxia in the animal, requiring it to be an objective, quick and effective maneuver (3, 4, 19, 21). This is an unprecedented study demonstrating that acupuncture is a resuscitation procedure with a rapid response and evolution of vital parameters in neonatal puppies, contributing to clinical improvement and a higher survival rate.

## 5 Conclusion

The VG26 and K1 acupuncture points are effective as resuscitation maneuvers in neonatal puppies, leading to an improvement in the respiratory pattern and an increase in heart and respiratory rates, and can be used in immediate neonatal care at birth. The neonatal response to the use of VG26 or K1 points does not differ from each other; both have similar effects and provide clinical improvement and stabilization of HR, RR and the Apgar score, providing alternative procedures in the resuscitation protocol.

## Data Availability

The original contributions presented in the study are included in the article/Supplementary material, further inquiries can be directed to the corresponding author.

## Supplementary material

VIDEO 1 Needling at the VG26 acupoint in a neonatal dog, demonstrating pistoning movement.

VIDEO 2 Needling of the K1 acupoint in the left pelvic limb of a neonatal dog, with a pistoning movement.
